# A deep neural network for general scattering matrix

**DOI:** 10.1515/nanoph-2022-0770

**Published:** 2023-04-03

**Authors:** Yongxin Jing, Hongchen Chu, Bo Huang, Jie Luo, Wei Wang, Yun Lai

**Affiliations:** National Laboratory of Solid State Microstructures, School of Physics, and Collaborative Innovation Center of Advanced Microstructures, Nanjing University, Nanjing 210093, China; Information Hub, Hong Kong University of Science and Technology (Guangzhou), Guangzhou 511458, China; School of Physical Science and Technology, Soochow University, Suzhou 215006, China; Hong Kong University of Science and Technology, Hong Kong, China

**Keywords:** deep neural network, inverse problem, scattering matrix

## Abstract

The scattering matrix is the mathematical representation of the scattering characteristics of any scatterer. Nevertheless, except for scatterers with high symmetry like spheres or cylinders, the scattering matrix does not have any analytical forms and thus can only be calculated numerically, which requires heavy computation. Here, we have developed a well-trained deep neural network (DNN) that can calculate the scattering matrix of scatterers without symmetry at a speed thousands of times faster than that of finite element solvers. Interestingly, the scattering matrix obtained from the DNN inherently satisfies the fundamental physical principles, including energy conservation, time reversal and reciprocity. Moreover, inverse design based on the DNN is made possible by applying the gradient descent algorithm. Finally, we demonstrate an application of the DNN, which is to design scatterers with desired scattering properties under special conditions. Our work proposes a convenient solution of deep learning for scattering problems.

## 1 Introduction

In the past decade, deep neural network (DNN) has developed rapidly in the modern computational science and engineering such as natural language processing [1], computer vision [2], object classification [3], and regression problems [4], etc. DNN is similar to the structure of the neurons in human’s brain; the information can be processed and transmitted amongst the nodes. By optimizing the parameters in neurons, DNN owns the capability of self-teaching. Due to its strong nonlinear fitting ability, this data-driven approach has also been applied to nanophotonics. The representative examples include the design of metamaterials and metasurfaces [5], [6], [7], [8], [9], [10], [11], [12], [13], [14], holographic imaging [15], diffractive deep neural network [16], [17], [18], etc. It is generally anticipated that the method of DNN will play an increasingly important role in the general design of nanophotonics.

However, so far, the application of DNN to scattering problems is still very limited. The scattering of waves is one of the most fundamental and important phenomena in wave physics [19], [20], [21], [22], [23], [24], [25]. Most of previous studies were limited to scatterers with high symmetry like cylinders and spheres [26], [27], [28], [29], [30], [31], [32], [33], [34], [35], where the scattering matrix has only diagonal terms and analytical solutions. Nevertheless, for general scatterers without high symmetry, the scattering matrix has off-diagonal terms, which represent the coupling between the channels of different angular momenta. More critically, the scattering matrix for general scatterers without high symmetry does not have any analytical solutions, thus it can only be calculated numerically, e.g., by using finite element method (FEM) or finite difference time domain method (FDTD), which are much more demanding on the computing time and resources. Especially, inverse design has always been a hard task for the conventional numerical methods. Although some of the previous studies have studied scatterers with more diversified shapes via field measurement [28, 36], [37], [38], [39], [40], [41], [42], [43], [44], [45], [46], [47], [48], [49], [50], none of them has directly obtained the scattering matrices of the scatterers, which is vitally important for scattering analysis and design.

In this paper, we propose a DNN to obtain the scattering matrix of a scatterer without symmetry. The well-trained DNN can calculate the scattering matrix at high-speed thousands of times faster than that of FEM solvers. Although the physical laws like energy conservation, time reversal and reciprocity are not set as conditions in the scattering matrix, they are inherently satisfied in the calculation by the DNN. More importantly, the inverse design is enabled by applying the gradient descent (GD) algorithm into the same DNN, which means that the geometry of the scatterer can be obtained when the scattering matrix is given. Finally, we show that with the help of the DNN, many design tasks involved with scatterings can be accelerated, such as designing the scatterers with desired scattering properties under special conditions. The largely enhanced efficiency and flexibility by the DNN indicate that this approach could have large impact in solving scattering problems in nanophotonics.

## 2 Methods

### 2.1 Scattering matrix

Without loss of generality, we consider two dimensional optical systems where the electromagnetic field satisfies Helmholtz equation, the incident and scattered waves are expressed as the linear superposition of Bessel and Hankel functions with different orders. Here, we consider the transverse electric polarization with the electric field polarized along the *z*-direction, the electric field can be expressed as the linear combination of Bessel functions (incident) and Hankel functions of the first kind (scattered):

(1) $$\begin{array}{c} E\left(r,\theta \right)=\overset{+\infty }{\underset{n=-\infty }{\sum }}\left[\alpha_{n}J_{n}\left(kr\right)e^{in\theta }+\beta_{n}H_{n}^{\left(1\right)}\left(kr\right)e^{in\theta }\right], \end{array}$$

where *E* is the electric field, $k=\sqrt{\varepsilon }k_{0}$ , *ɛ* is the relative dielectric constant of medium and *k*_0_ is the wave vector in vacuum. *n* is an integer which denotes the order of Bessel function and Hankel function, besides, *n* can also be regarded as the orbital angular momentum of the incident/scattered waves. *α*_*n*_ and *β*_*n*_ are the coefficients of the incident and scattered waves, regarding to angular momentum *n*, respectively. An incident plane wave propagating in the *x*-direction can be written as $e^{i}\mathrm{kx}=\sum_{n=-\infty }^{+\infty }i^{n}J_{n}\left(kr\right)e^{in\theta }$ .

The coefficient of scattered waves, *β*_*n*_, is determined by both *α*_*n*_ and the scattering characteristics of the object under study, thus the elements of scattering matrix can be defined as

(2) $$\begin{array}{c} t_{nm}=\frac{\beta_{n}}{\alpha_{m}}, \end{array}$$

and the scattering matrix is $\overset{↔}{T}=\left\{t_{nm}\right\}$ .

Due to the independence between different angular momenta, the coefficients can be obtained if the incident field is known, i.e., the scattered coefficients can be calculated as

(3) $$\begin{array}{c} \beta_{n}=\frac{\oint E_{s}\cdot e^{-in\theta }d\theta }{2\pi H_{n}^{\left(1\right)}\left(kr_{0}\right)}, \end{array}$$

where *E*_*S*_ = *E* − *E*_inc_ is the scattered field, the numerator is a circular integral over theta on a circle of radius *r*_0_. And the scattering matrix can thus be acquired according to Equation (2). The main computational approach is similar in the three-dimensional case, while the basic functions are more complicated thus the matrix dimensions are higher. The details of derivative process in 3D case are shown in Supplementary Material.

Once the scattering matrix is obtained, the scattering problem is solved and the total field is determined by Equation (1). However, we note that there are also some relations between the elements of the scattering matrix, implying the influence of some fundamental physical laws. For example, energy conservation can be expressed as:

(4) $$\begin{array}{c} \underset{m}{\sum }{\left|t_{mn}\right|}^{2}+Re\left(t_{nn}\right)=0, \end{array}$$

time reversal is:

(5) $$\begin{array}{c} \underset{m}{\sum }4{\left(-1\right)}^{m-n}t_{mn}^{*}t_{-n,-m}+2\left(t_{nn}^{*}+t_{-n,-n}\right)=0, \end{array}$$

and reciprocity is:

(6) $$\begin{array}{c} t_{-n,-m}={\left(-1\right)}^{n-m}t_{mn}. \end{array}$$

Actually, when reciprocity is satisfied, energy conservation and time reversal are equal conditions. A simple proof is shown in the Supplementary Materials.

### 2.2 Deep neural network

**Figure 1:**
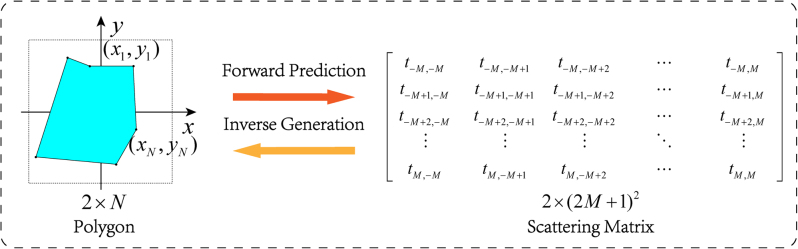
The schematic diagram of the conservation between polygon coordinates and scattering matrix (with complex elements).

We define a polygon as the scatterer with complicated shape. The polygon is determined by the coordinates of its vertices, the degree of freedom (DoF) is 2 × *N*, where *N* is the number of sides of the polygon. The corresponding scattering matrix is a (2*M* + 1) × (2*M* + 1) complex matrix, so the DoF is 2 × (2*M* + 1)^2^. The angular momentum *n* and *m* in the scattering matrix both can change from −*M* to *M*, where *M* is a finite integer for the scatterer of finite size, because the response to high order angular momentum is usually small and can be ignored. This conversion process is showed in Figure 1. Apparently, the off-diagonal terms introduced by low symmetry has significantly increased the dimensionality of the scattering matrix. Such a high dimensional problem cannot be tackled by analytical methods and is therefore suitable for the method of DNN.

**Figure 2:**
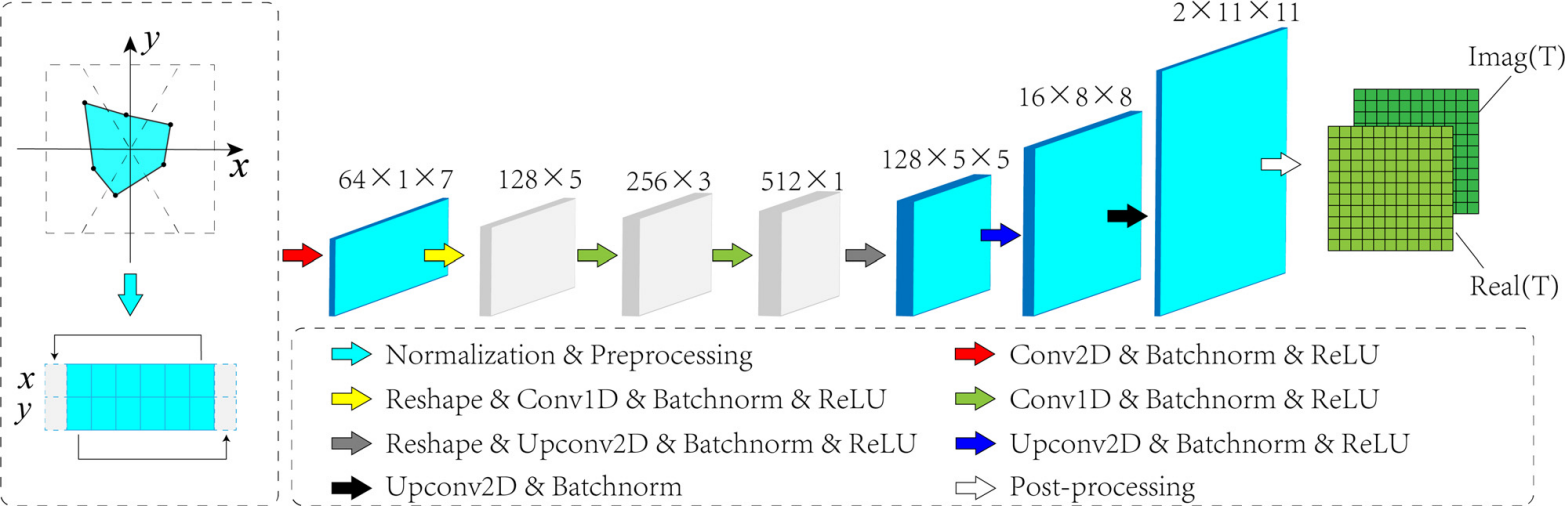
The structure of DNN. The different operations are indicated by arrows with different colors and identified with legends below.

The construction of the polygon is shown in Figure 2. To impose restrictions on size of the input polygon, the coordinates are limited in a rectangular region. This region is divided into *N* areas according to the equal division of the angle at the coordinate origin. By picking a random point in every area, connecting the *N* points and a polygon can be generated.

The structure of DNN used in this work is demonstrated in Figure 2. The inputs are normalized. In order to preserve the connecting characteristics of these vertices, we copy the coordinate of the first vertex and placed it after the last vertex, and vice versa. It turns out that this trick can remarkably optimize the training results of the DNN. The structure of DNN are similar to a U-Net, where the 2D and 1D convolutional layers have completed the down-sampling process to extract the hidden abstract information, then the transposed convolutional layers have recovered the information to construct a corresponding scattering matrix, i.e., up-sampling. The two channels of output represent the real and imaginary part of scattering matrix. The active function is ReLU, loss function is mean square error (MSE) and optimization algorithm is stochastic gradient descent (SGD).

### 2.3 Inverse design process

The well-trained DNN calculates the forward process in a fast and accurate way, which means that although the relation between coordinates and elements of the scattering matrix is complicated that we cannot write down the function explicitly, the DNN have indeed established a solvable function. Based on this property, inverse design can be realized by applying GD. The basic operation is as shown in Figure 4(a).

In the inverse design task, the parameters of DNN are pre-trained and fixed. Firstly, the DNN will calculate the scattering matrix of a random initial polygon, as well as the difference between target matrix with the predicted matrix. The difference is a function of coordinates only. To reduce the difference, the GD algorithm is applied to optimize the coordinates. The polygon will approach the correct solution gradually after iterations.

Actually, the final inverse result is related to the choice of the initial polygon, thus we add randomness into the original coordinates and perform multiple calculations. Although the results might converge to different polygons, they have similar size and shape, as well as scattering matrix.

## 3 Results

### 3.1 Forward process and application

Consider a polygon with *N* = 6 whose size is around 0.4*λ*_0_, where *λ*_0_ is the wave length in free space, the material of the polygon is set as silicon dioxide with a dielectric constant of 3.9. For scatterers of this size, the scattering matrix converges rapidly along with the order of angular momentum, i.e., the low-order elements are the dominant terms in the matrix, and thus we describe it by a 11 × 11 matrix, i.e., *M* = 5. Besides, the DNN is able to predict scattering matrices of different dielectric constant, even lossy media, when we add more training data and fine-tune the structure, see Supplementary Material for details.

**Figure 3:**
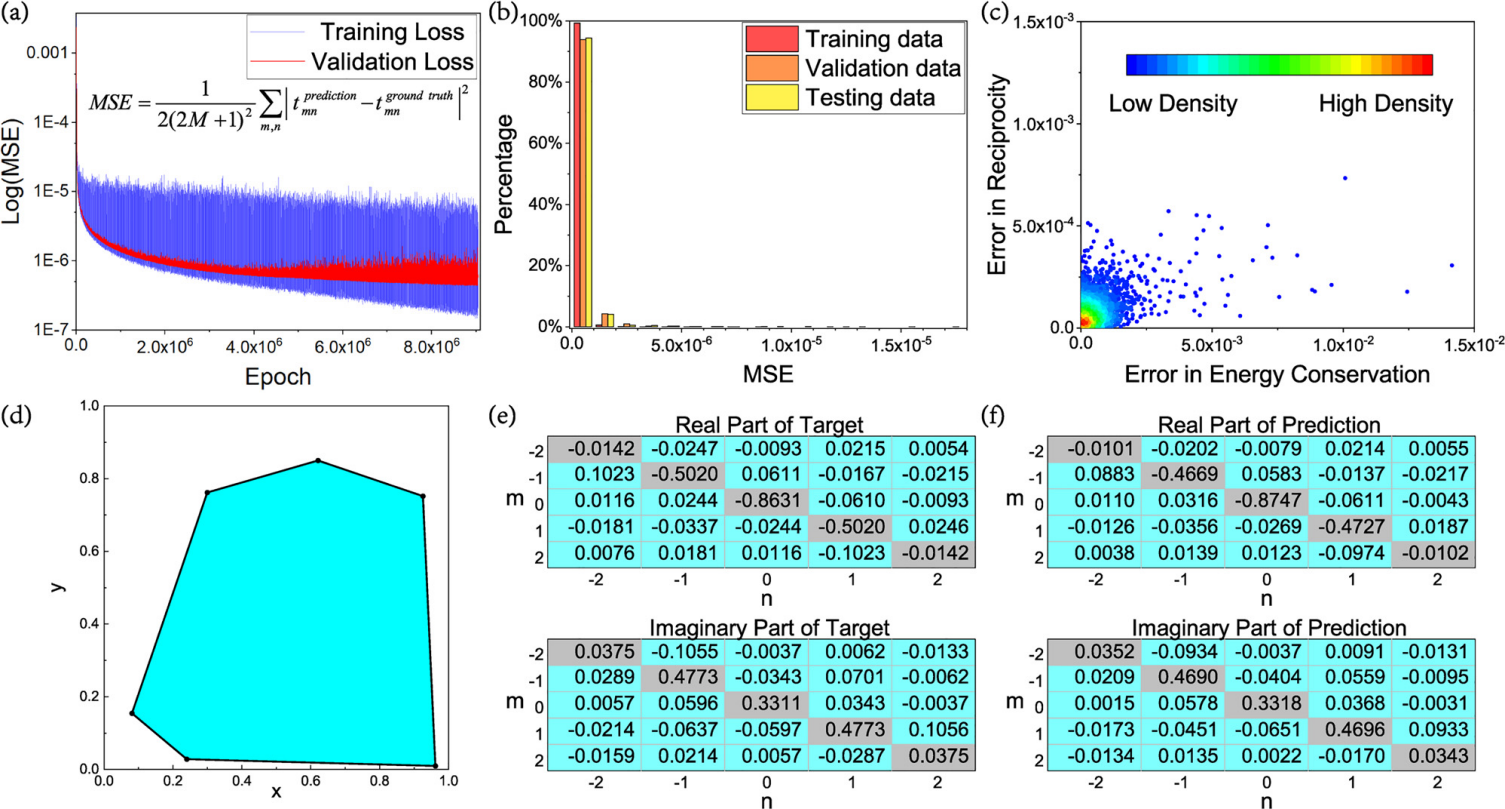
Results of forward process. (a) Training loss (blue) and validation loss (red) over epochs during training. The loss function is MSE which is also denoted. (b) The loss distribution histogram of training data (red), validation data (green) and testing data (yellow). (c) The distribution of error in energy conservation (*x* axis) and reciprocity (*y* axis) of 10^6^ random polygons’ predictions, the density is indicated as red to blue from large to small. (d) The polygon with LOSS = 1.75 × 10^−5^, which is the sample with the greatest error. (e) Real part (above) and imaginary part (below) of target scattering matrix of (d). (f) Real part (above) and imaginary part (below) of predicted scattering matrix of the sample in (e).

To train the forward DNN, the dataset is composed of 36,000 training data, 4000 validation data and 4000 testing data. The training data are obtained by using the commercial finite element solver COMSOL Multiphysics with Equation (3), the specific configuration of the model is described in details in the Supplementary Material. From Figure 3(a), we find that both training loss (blue line) and validation loss (red line) decrease as the training process goes on and finally maintain in a low value. During training, weight decay is set as 10^−3^ to suppress overfitting efficiently. To assess the accuracy of the trained DNN, the three kinds of data are input into the model to calculate the MSE between predicted scattering matrix and ground truth, the distribution histogram of data is plotted in Figure 3(b) with different colors. The proportion of loss lower than 1 × 10^−6^ is more than 93% for all three kinds of data; meanwhile the percentage rises to 99% when considering the loss lower than 4 × 10^−6^. The biggest MSE in the dataset equals to 1.75 × 10^−5^, the corresponding polygon is plotted in Figure 3(d). And the real and imaginary parts of its scattering matrix are displayed in the upper and lower parts of Figure 3(e), respectively. Since the higher-order terms are very close to zero, we only show the main parts, i.e., the low-order terms. The corresponding prediction is shown in Figure 3(f), actually, the matrixes are quite similar, verifying the accuracy of DNN.

**Figure 4:**
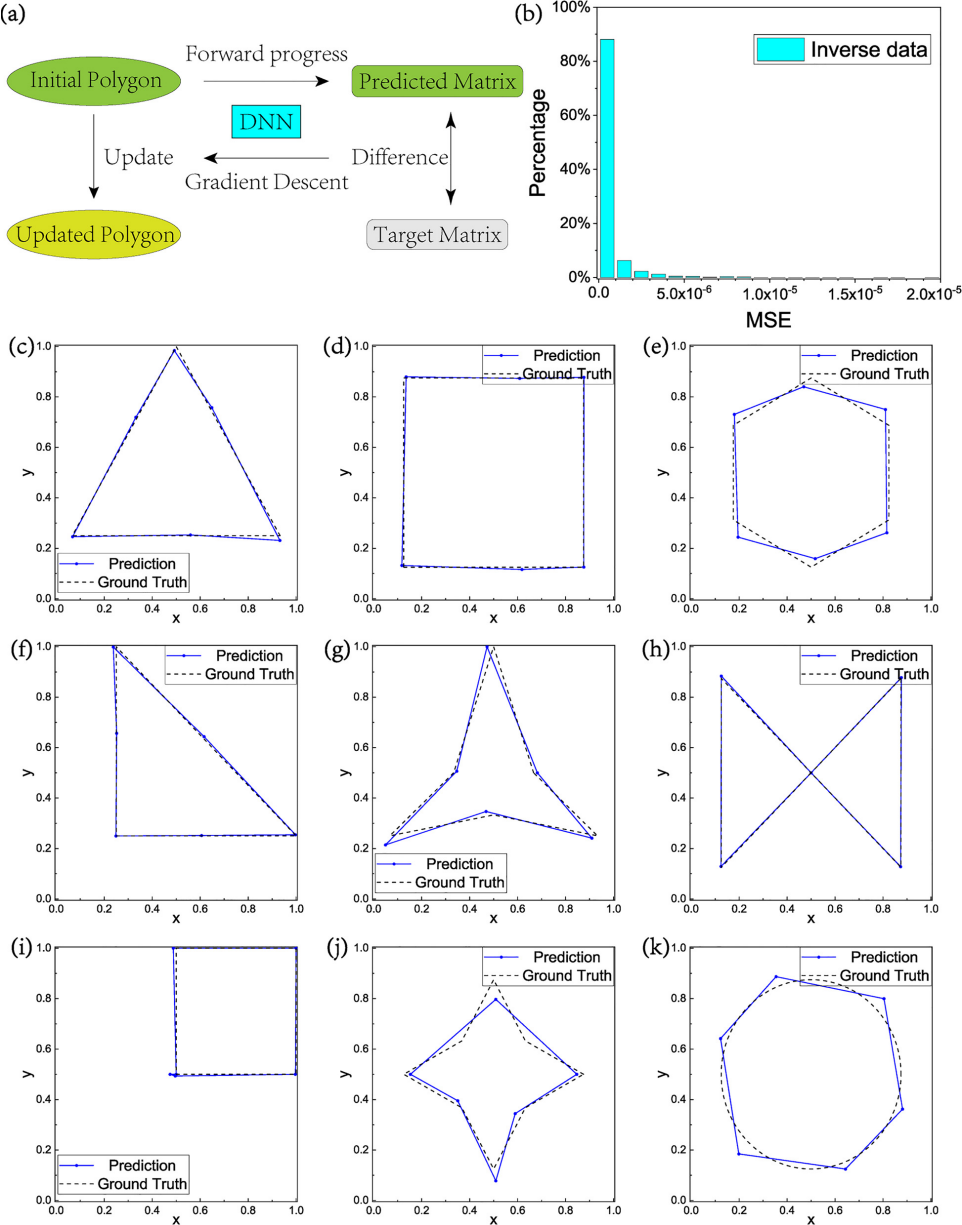
Result of the inverse process. (a) The schematic diagram of the inverse process. (b) The loss distribution histogram between target and inverse scattering matrix. (c–k) The predicted polygons (blue solid lines) compared with the target pattern (black dash lines).

More importantly, the speed of DNN to calculate the scattering matrix is significantly higher than those of conventional numerical methods. Here, we made a comparison with the commercial FEM solver COMSOL Multiphysics. It turns out the DNN has a speed 7488 times faster than that of COMSOL in average. The details are shown in Supplementary Materials.

Although we haven’t fed the DNN with any physical laws, the DNN have the ability to discover some fundamental principles by self-teaching. This is because, as a data-driven method, neural networks have strong nonlinear fitting capabilities, and therefore can extract such patterns when the training data has similar characteristics. All the polygons for training satisfy reciprocity and energy conservation, which can be expressed by the special relationships between special matrix elements. A hundred thousand randomly generated polygons are input into DNN and the corresponding output are analyzed, the errors in the two fundamental laws of physics are expressed as a point in the two-dimensional coordinate system, which is shown in Figure 3(c). The density of points is distinguished by different colors, most of the points distribute around the origin, which means the errors are both small. The result proves that results obtained from the DNN have inherently satisfied the two physical principles by mining the common features of the training data. Meanwhile, utilizing the known fundamental laws during training process can also improve the DNN accuracy, which draws a lesson form the physical informed neural networks (PINNs) [31, 32], we have discussed the details of the results in Supplementary Material.

### 3.2 Inverse design results

The scattering matrixes of 4000 testing data are put into the inverse process as ground truth to assess the accuracy rate. DNN had generated corresponding polygon coordinates, and then we recalculated the exact scattering matrix of these results with FEM. The MSE between the targeted and recalculated scattering matrices are demonstrated in Figure 4(b). 88% of the inverse results exhibits loss below 10^−6^, and the proportion is 98% when we loosen the restriction to 5 × 10^−6^. The histogram is an absolute proof of DNN’s ability to inverse generating.

To further demonstrate the effects intuitively, we put several regular polygons’ scattering matrixes calculated by FEM into the DNN. The predicted polygons are plotted with blue solid lines and points in Figure 4(c)–(k), as a contrast, the ground truth polygons are also shown with black dash lines in the same pictures. The prediction fits well with ground truth. Besides, although Figure 4(i)–(k) actually are beyond the domain of training data, inverse process has obtained proximate solution as far as possible. Although there are some deviations in the details of the shapes, the scattering matrix elements of these objects are almost the same as the original shape. For example, the MSE between the predicted hexagon and origin circle in Figure 4(k) equals to 4.2 × 10^−7^. Actually, from Figure 4(k), it is seen that the overlapping region of the original and inversely designed scatterers is over 96% in area. These results indicate that our DNN possesses ability of discovering information about scattering characteristics from the shape of scatterer.

### 3.3 Applications

**Figure 5:**
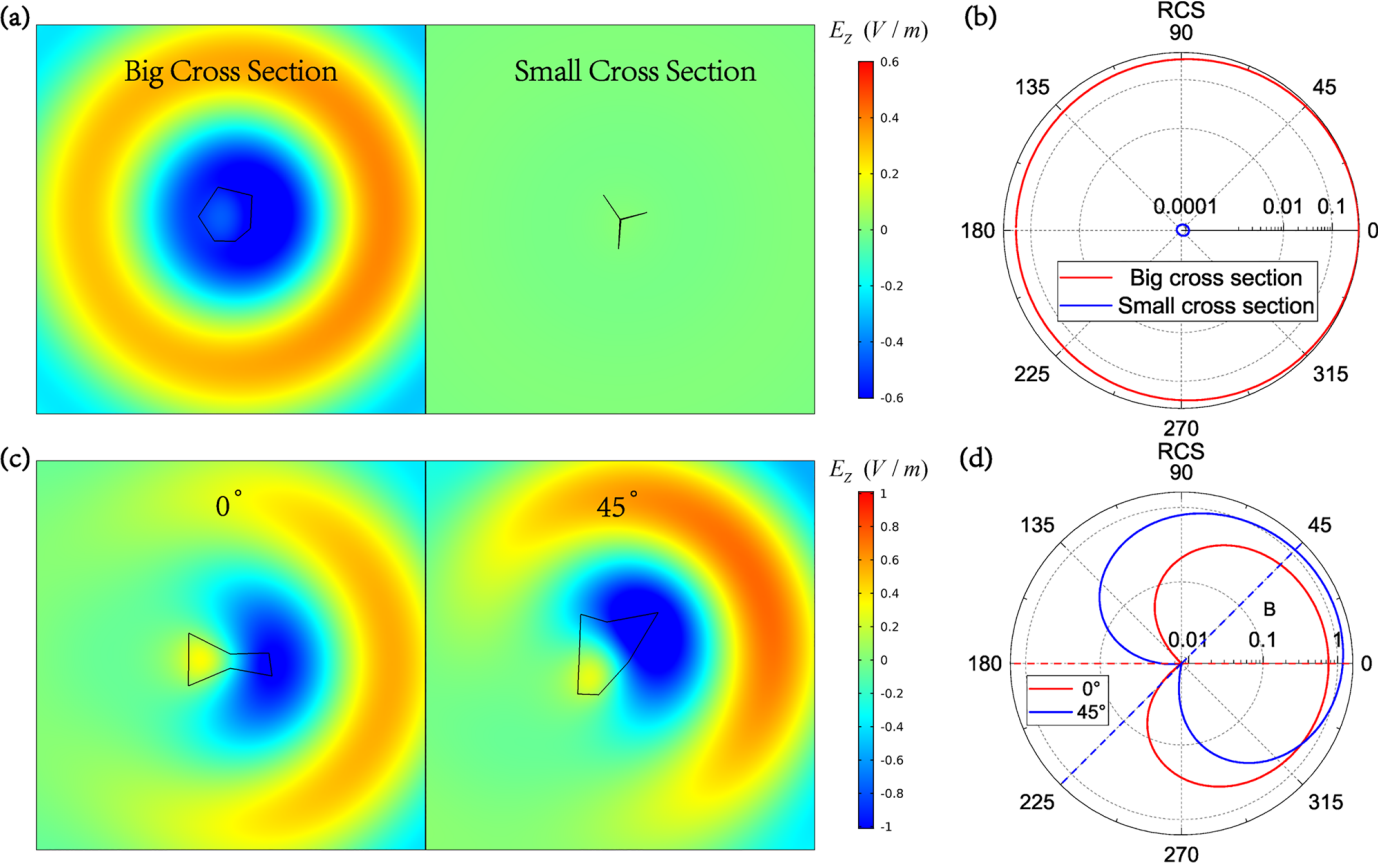
More applications of the DNN. (a) The scattered electric field distribution of polygons with big cross section (left) and small cross section (right) while they have the same perimeter. (b) Radar cross section of the polygons in (a), the big cross section and small cross section are denoted by red line and blue line, respectively. (c) The scattered electric field distribution of polygons with the radiation direction toward 0° (left) and 45° (right). (d) Radar cross section of the polygons in (c), the radiations toward different angles are denoted by different colors.

There are many practical applications of the DNN, for example, we can traverse a mass of polygons at a high speed to find the shapes with maximal and minimal scattering cross section. Here we calculated 5 × 10^5^ hexagons with the same perimeter of 2 using the pre-trained DNN to find the target polygons. Simulating the same quantity of samples by FEM needs to cost about a month, but with DNN the task is finished in 811 s. The results are quite interesting. The hexagons with the maximal and minimal scattering cross sections are similar to a circle and a collection of thin branches, respectively. When they are illuminated by a plane wave propagating along the *x*-axis, the scattered electric fields are plotted in the left and right image of Figure 5(a), respectively. Obviously, the near scattered fields are dramatically different. Figure 5(b) shows the corresponding radar cross section (RCS), the difference is about 3 orders of magnitude.

With the pre-trained DNN, it is also possible to find out the polygon that has the specific angle of radiation. By processing the scattering matrix elements, the far-field scattering amplitude at every angle can be calculated, just as described in Supplementary Materials, thus we can obtain the ratio of the amplitude at *θ* to *θ* + *π*. Radiation will be concentrated around *θ* when the ratio is big enough. Another random 5 × 10^5^ hexagons are input into the DNN to search for the polygon with the biggest ratio when *θ* is equal to 0 and 45°. The electric fields are plotted in Figure 5(c) and the corresponding RCS are shown in Figure 5(d). Near field distribution is quite different and the center of the RCS major lobes is indeed located around the desired *θ* (0 and 45°) and there is a minimum value at *θ* + *π* according to the results. We note that the lobe has a large width because the polygon is in sub-wavelength scale. The results in Figure 5(c) match well with those in Figure 5(d).

## 4 Discussion

Scattering matrix can represent the scattering characteristics of scatterers in a concise way. The high dimensionality of the scattering matrix of scatterers without high symmetry makes it challenging to tackle with conventional numerical methods, but a perfect problem for DNN. Here, we have considered the case of hexagons composed of a single material. But this limitation can be easily relieved by applying more general models. For instance, the model of a distribution of dielectric constant in grids, which magnifies the DoF of input polygon [36], [37], [38], [39], [40], [41], [42], [43], [44], [45], [46], [47], [48], [49], [50], [51], [52], is worth exploration in the future. Besides, the scattering matrices of similar polygons are approximate because the difference is deep subwavelength, which leads to non-uniqueness of the solution. To improve the accuracy to distinguish them, shorter wavelength could be considered. As a result, the scatterers will have larger response to the high order of angular momentum and the scattering matrices should be described with more terms, i.e., *M* needs to be bigger. Although these measures can improve the performance of DNN, they need more training data and more complicated network structure.

In summary, we have demonstrated a DNN that establishes the relation between the geometry of a dielectric hexagon to its scattering matrix. The scattering characteristics can be obtained by the well-trained DNN in a quick and concise way. The speed of the well-trained DNN is thousands of times faster than that of finite element solvers. Therefore, previous tasks of heavy computation are now possible. Furthermore, by applying GD algorithm, the same DNN can complete the inverse process with high accuracy. To the best of our knowledge, this is the first time that the general scattering matrix is obtained with a DNN. Our results indicate that the strategy of DNN for scattering matrix is a convenient and efficient way to solve general scattering problems.

## Supplementary Material

Supplementary Material Details
