# Supplementary material for: A deep neural network for general scattering matrix
Source: Nanophotonics. 2023 Apr 3;12(13):2583–91. doi: 10.1515/nanoph-2022-0770 (PMC11501312; doi:10.1515/nanoph-2022-0770)
Supplement: Supplementary file 1 — Supplementary Material Details [file j_nanoph-2022-0770_suppl_001.pdf]

Yongxin Jing, Hongchen Chu, Bo Huang, Jie Luo, Wei Wang*, Yun Lai*

Supplementary of A deep neural network for general scattering matrix

1 Some fundamental properties of the scattering matrix

1.1 Energy conservation

The polygons we discussed in the article is lossless, so the optical system satisfies energy conservation, i.e., the net energy flow that passes through a closed curve equals to zero. Considering a circle A whose center is the origin of coordinates and the radius is large enough such that the scatterer is surrounded. The net energy flow that passes though A is

$$W_a = -\oint \vec{S} \cdot \hat{e_r} dA, \tag{1}$$

where $\vec{S}$ is the Poytning vector, $\hat{e_r}$ denotes the unit vector along the radial direction, the loop path integral is over A. Put the expression of Poytning vector into the formula, we get

$$\vec{S} \cdot \hat{e_r} = -\frac{1}{2}\mathrm{Re}(E_z \cdot H_\theta^*). \tag{2}$$

E_z and H_θ are radial component of electric field and angular component of magnetic field, respectively. According to Maxwell's equations, the magnetic field can be deduced from the electric field. Consider the situation that incident wave is denoted by $J_n(kr)e^{in\theta}$, and the scattering wave is $\sum_m t_{mn} H_m^{(1)}(kr)e^{im\theta}$, the magnetic field is expressed as

$$\frac{\omega\mu_0}{i} H_\theta = -\frac{1}{r}\frac{\partial}{\partial r}E_z = -\frac{1}{r}\left(\frac{\partial}{\partial r}J_n(kr)e^{in\theta} + \sum_m t_{mn}\frac{\partial}{\partial r}H_m^{(1)}(kr)e^{im\theta}\right), \tag{3}$$

where ω is the frequency and μ_0 is permeability of free space. To simplify the problem, we set $\mathrm{kr} = \mathrm{x} \to \infty$, the asymptotic relation of Bessel functions is

$$J_n(x) \propto \sqrt{\frac{2}{\pi x}}\cos\left(x - \frac{n\pi}{2} - \frac{\pi}{4}\right), \tag{4}$$

$$\mathrm{H}_n^{(1)}(x) \propto \sqrt{\frac{2}{\pi x}}e^{i\left(x-\frac{n\pi}{2}-\frac{\pi}{4}\right)}, \tag{5}$$

the recurrence relation is introduced to eliminate the partial derivation:

$$Z_{n-1} - Z_{n+1} = 2Z_n'(x), \tag{6}$$

the equation is workable for all kinds of Bessel functions. Due to the integral path is a circle, the integral can be rewritten as

***Corresponding author: Yun Lai,** National Laboratory of Solid State Microstructures, School of Physics, and Collaborative Innovation Center of Advanced Microstructures, Nanjing University, Nanjing 210093, China, E-mail: laiyun@nju.edu.cn; **Wei Wang,** Information Hub, Hong Kong University of Science and Technology (Guangzhou), Guangdong 510000, China, E-mail: weiwcs@ust.hk
Yongxin Jing, National Laboratory of Solid State Microstructures, School of Physics, and Collaborative Innovation Center of Advanced Microstructures, Nanjing University, Nanjing 210093, China, E-mail: yxjing@smail.nju.edu.cn
Hongchen Chu, National Laboratory of Solid State Microstructures, School of Physics, and Collaborative Innovation Center of Advanced Microstructures, Nanjing University, Nanjing 210093, China, E-mail: chuhongchen@nju.edu.cn
Bo Huang, Information Hub, Hong Kong University of Science and Technology (Guangzhou), Guangdong 510000, China, E-mail: bhuangas@connect.ust.hk
Jie Luo, School of Physical Science and Technology, Soochow University, Suzhou 215006, China, E-mail: luojie@suda.edu.cn

$$\oint dA = \int_0^{2\pi} rd\theta\,. \tag{8}$$

The result can be derived from the simultaneous equations above:

$$W_a = 0 \Leftrightarrow \sum_m |t_{mn}|^2 + Re(t_{nn}) = 0. \tag{9}$$

1.2 The expression of cross section

The cross section is defined as the ratio of the scattered energy with the incident energy, the derivative process is quite similar to 1.1. The scattered energy is

$$W_s = -\oint \vec{S}_{sc} \cdot \hat{e_r} dA\,, \tag{10}$$

where $\vec{S}_{sc}$ denotes Poytning vector of the scattered wave. In the same way, we introduce Maxwell's equations:

$$E_{sc} = \sum_m \beta_m H_m^{(1)}(kr) e^{im\theta}\,, \tag{11}$$

$$\vec{H}_{sc} = \frac{i}{\omega\mu_0} \nabla \times \vec{E}_{sc}. \tag{12}$$

After some derivation, we get

$$W_s = \frac{2}{\omega\mu_0} \sum_m |\beta_m|^2\,. \tag{13}$$

When the scattering matrix and incident wave are known, the scattering coefficient is $\beta_m = \sum_n t_{mn}\alpha_n$. Considering the incident plane wave propagating along x axis, the incident coefficient is $\alpha_n = i^n$, so the cross section is proportional to $\sum_m |\sum_n i^n t_{mn}|^2$.

1.3 Another kind of the scattering matrix

Although the incident wave is expressed as the superposition of Bessel functions in our research, there is another common definition where the incident wave is described by the first kind of Hankel functions.

Reviewing the calculating process of the first kind of scattering matrix, the incident wave is:

$$E_{inc} = J_n(kx)e^{in\theta} = \frac{1}{2}\left[H_n^{(1)}(kr) + H_n^{(2)}(kr)\right]e^{in\theta} = E'_{inc} + \frac{1}{2}H_n^{(1)}(kr)e^{in\theta}. \tag{14}$$

The superscript denotes the incident wave in the second definition of the scattering matrix. The corresponding scattered wave is:

$$E_{sc} = E_{total} - E_{inc} = \sum_m t_{mn} H_m^{(1)}(kr) e^{im\theta}\,, \tag{15}$$

$$E'_{sc} = E_{total} - E'_{inc} = \sum_m \frac{1}{2} t'_{mn} H_m^{(1)}(kr) e^{im\theta}\,, \tag{16}$$

$$= \sum_m t_{mn} H_m^{(1)}(kr) e^{im\theta} + \frac{1}{2} H_n^{(1)}(kr) e^{in\theta}. \tag{17}$$

The terms are equal when the subscript is the same, then we get the relation between the original and new scattering matrices as:

$$T' = 2T + I, \tag{18}$$

where I is unit matrix.

1.4 Reciprocity

Considering the second definition, the incident wave is set as $\alpha'_n H_n^{(2)}(kr)e^{in\theta}$ and one channel of the scattered wave is $\beta'_m H_m^{(1)}(kr)e^{im\theta}$. After reciprocity conversion, the incident wave is expressed as

$$\alpha'_n H_m^{(2)}(kr)e^{-im\theta} = (-1)^m \alpha'_n H_{-m}^{(2)}(kr)e^{-im\theta}, \tag{19}$$

and the corresponding scattering channel is

$$\beta'_m H_n^{(1)}(kr)e^{-in\theta} = (-1)^n \beta'_m H_{-n}^{(1)}(kr)e^{-in\theta}. \tag{20}$$

Meanwhile, according to the definition of the scattering matrix,

$$t'_{mn} = \frac{\beta'_m}{\alpha'_n}, \tag{21}$$

$$t'_{-n,-m} = \frac{(-1)^n \beta'_m}{(-1)^m \alpha'_n} = (-1)^{n-m} t'_{mn}. \tag{22}$$

And the original scattering matrix should satisfy the same relation according to equation (18):

$$t_{-n,-m} = (-1)^{n-m} t_{mn}. \tag{23}$$

1.5 Time reversal

The proof of time reversal is similar to reciprocity. The incident wave is denoted by $\alpha'_n H_n^{(2)}(kr)e^{in\theta}$ and the scattering wave is $\sum_m \beta_m H_m^{(1)}(kr)e^{im\theta}$, after time reversal, we should take the conjugate of the field:

$$E'_{inc} = \sum_m \beta'^*_m H_m^{(2)}(kr)e^{-im\theta} = \sum_m (-1)^m \beta'^*_m H_{-m}^{(2)}(kr)e^{-im\theta}, \tag{24}$$

$$E'_{sc} = \alpha'^*_n H_n^{(1)}(kr)e^{-in\theta} = (-1)^n \alpha'^*_n H_{-n}^{(1)}(kr)e^{-in\theta}. \tag{25}$$

According to the definition of the scattering matrix, we have:

$$\beta'^*_m = \alpha'^*_n \cdot t'^*_{mn}, \tag{26}$$

$$\sum_m (-1)^m \beta'^*_m\, t'_{-n,-m} = (-1)^n \alpha'^*_n. \tag{27}$$

Thus, we get:

$$\sum_m (-1)^{m-n} t'^*_{mn}\, t'_{-n,-m} = 1. \tag{28}$$

Besides, if the system satisfies reciprocity, the equation is reduced to:

$$\sum_m (-1)^{m-n} t'^*_{mn}\, t'_{-n,-m} = \sum_m (-1)^{m-n} t'^*_{mn}\,(-1)^{m-n} t'_{mn} = \sum_m |t'_{mn}|^2 = 1. \tag{29}$$

In this situation, time reversal and energy conservation are equal if we put equation (18) into formula (29).

2 Far-field amplitude at a specific angle

The scattered field is the linear superposition of different Hankel functions. By applying the progressive form when $r \to \infty$, the far-field scattering is:

$$\lim_{r\to\infty} E_{sc} = \sum_m \beta_m e^{im\theta} \lim_{r\to\infty} H_m^{(1)}(kr) \propto \sum_m \beta_m e^{im\theta} \sqrt{\frac{2}{\pi kr}} e^{i\left(kr - \frac{m\pi}{2}\right)}. \quad (29)$$

The amplitude at a specific angle θ_0 is proportional to:

$$A(\theta_0) \propto \left| \sum_m \beta_m \sqrt{\frac{1}{kr}} e^{im\left(\theta_0 - \frac{m\pi}{2}\right)} \right| = \left| \sum_n \sum_m \alpha_n \sqrt{\frac{1}{kr}} e^{im\left(\theta_0 - \frac{m\pi}{2}\right)} \right|. \quad (30)$$

3 The computation speeds of FEM and DNN

When calculating scattering matrix with finite element method (FEM), e.g., COMSOL Multiphysics, the simulation software will grid the object to calculate the electromagnetic field at every block, and the accuracy is determined by the mesh density. Here, we have applied the mesh elements whose sizes are between $\lambda_0/50$ and $\lambda_0/10$, this configuration is also used to calculate the training data. The formula of Equation (3) in the main text is used to calculate scattering matrix, where the calculation is also made in COMSOL.

We have generated 10^4 randomly polygons, and put them into COMSOL and well-trained DNN separately to calculate scattering matrices. COMSOL has cost 52645.71 seconds, which means the average speed is 5.26 seconds. However, DNN only costs 7.03 seconds, the average speed is 7.03×10^{-4} seconds. The data shows that DNN's speed is about 7488 times faster than that of COMSOL, demonstrating the excellent performance of the DNN.

4 The derivative process of three-dimensional case

Scattering matrix method is a universal approach to solve scattering problems which is appropriate for both two dimensional and three-dimensional situations. We have demonstrated the 2D scattering matrix, now we would like to supplement the derivation of 3D scattering matrix.

Firstly, we will discuss about the vector spherical wavefunctions (VSWFs):

$$\mathbf{M}_{nm}^{(1,2)}(kr) = N_n h_n^{(1,2)}(kr) \mathbf{C}_{nm}(\theta,\varphi), \quad (31)$$

$$\mathbf{N}_{nm}^{(1,2)}(kr) = \frac{h_n^{(1,2)}(kr)}{krN_n} \mathbf{P}_{nm}(\theta,\varphi) + N_n \left[h_{n-1}^{(1,2)}(kr) - \frac{n h_n^{(1,2)}(kr)}{kr} \right] \mathbf{B}_{nm}(\theta,\varphi), \quad (32)$$

where r, θ, φ denote the usual polar spherical coordinates, k is the wavenumber. $N_n = 1/\sqrt{n(n+1)}$ is normalization constant. $h_n^{(1,2)}(kr)$ are spherical Hankel functions of the first and the second kind, which describe the radial distributions of the waves. $\mathbf{C}_{nm}(\theta,\varphi)$, $\mathbf{P}_{nm}(\theta,\varphi)$ and $\mathbf{B}_{nm}(\theta,\varphi)$ are the vector spherical harmonics, they describe the angular distributions. However, the VSWFs above is divergence at the origin point, so we introduce another form of VSWFs:

$$\mathbf{M}_{nm}^{(3)}(kr) = \frac{1}{2}\left[\mathbf{M}_{nm}^{(1)}(kr) + \mathbf{M}_{nm}^{(2)}(kr)\right], \quad (33)$$

$$\mathbf{N}_{nm}^{(3)}(kr) = \frac{1}{2}\left[\mathbf{N}_{nm}^{(1)}(kr) + \mathbf{N}_{nm}^{(2)}(kr)\right], \quad (34)$$

they are the solutions of the standing waves in the three-dimensional space. Obviously, in this form, the radial distributions of the waves are described by the spherical Bessel functions $j_n(kr) = 1/2[h_n^{(1)}(kr) + h_n^{(2)}(kr)]$.

Then the incident and scattered electric fields are expressed as the sum of these VSWFs:

$$\mathbf{E}^{inc}(\mathbf{r}) = \sum_{n=1}^{\infty}\sum_{m=-n}^{n} a_{nm}\mathbf{M}_{nm}^{(3)}(kr) + b_{nm}\mathbf{M}_{nm}^{(3)}(kr), \tag{35}$$

$$\mathbf{E}^{sc}(\mathbf{r}) = \sum_{n=1}^{\infty}\sum_{m=-n}^{n} c_{nm}\mathbf{M}_{nm}^{(1)}(kr) + d_{nm}\mathbf{M}_{nm}^{(1)}(kr), \tag{36}$$

where a_{nm} and b_{nm} are incident coefficients, c_{nm} and d_{nm} are scattering coefficients. Same as the two-dimensional situation, we terminate the polynomial at $n = N_{max}$. The incident vectors are composed of all the a_{nm} and b_{nm}, which is of dimension $N_T = 2N_{max}(N_{max} + 2)$. The definition of scattering vectors is similar. Then the scattering matrix in three-dimensional case can be derived:

$$\begin{pmatrix} c_{nm} \\ d_{nm} \end{pmatrix} = \mathbf{T}\begin{pmatrix} a_{nm} \\ b_{nm} \end{pmatrix}. \tag{37}$$

The matrix is $N_T \times N_T$.

It's obvious that the number of elements in the 3D scattering matrices is much larger than the 2D scattering matrices, which means it is a harder question thus we need more training data. Besides, before training a neural network, we need to prepare the training data by FEM solver, as is well-known, the calculation speed of 3D situation is much slower than the 2D situation. Moreover, although the complexity in 3D problems is much greater, the fundamental approach and calculation steps are the same as 2D problems. Our main objective is to demonstrate how to establish the relation between scatterers and the scattering matrices by the deep neural network. For these reasons above, we have chosen the 2D situation as our main research topic, and we believe this is a cost-effective way to realize the goal.

5 The performance of DNN on lossy material

we have investigated the case where the permittivities of the scatterers are complex numbers, which means the media is lossy. The neural network (NN) we have introduced in the main text focuses on the media with permittivity equal to 3.9, so the input of the NN only contains shape information. Here, we modify the NN's input and structure slightly to enable it to learn the information of material. Specifically, we have connected the real and imaginary part of permittivity to the origin input as the second channel and input them together into the neural network (Fig.S1(a)). Correspondingly, we only need to modify the first convolutional layer of the NN. To train this modified NN, we have added another 40000 data points to the origin data, with 10000 data having a permittivity $3.9 + 0.1i$, 10000 data having a permittivity $3.9 + 0.5i$, and 5000 each having permittivity of $2, 3, 4$, and 5. These data are divided into training and validation data sets in a 9:1 ratio.

After training, we input 7000 testing data points, which contain different media. The NN has not seen these data during the training. The MSE distribution is shown in Fig.S1(b). The modified NN is able to predict the scattering matrix of different media, even lossy material. Although the performance of NN in predicting materials with high refractive indices is relatively poor, which is also in line with people's cognition, the MSE distribution is still satisfactory. If more training data of high refractive index materials are added, the performance may be further improved.

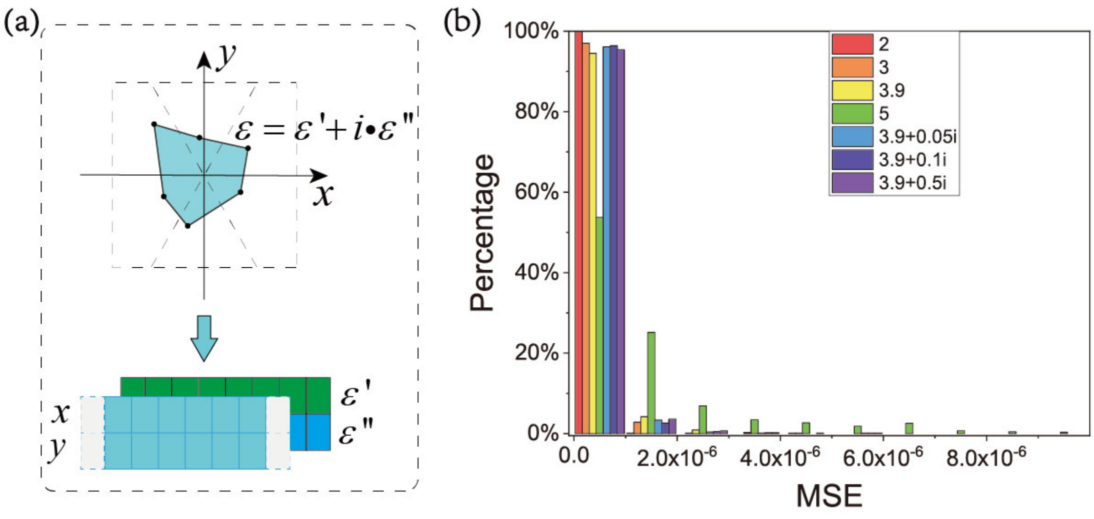

Fig.S1. (a) The changes in input format. (b) The MSE distribution of the testing data with different permittivities.

6 The configuration of FEM simulation

The FEM simulations are completed by COMSOL Multiphysics software, the model is built on a two-dimensional system. The incident wavelength is 0.5m, while the vertex coordinates of these polygons range from -0.1 to 0.1. These media are enclosed in a circular vacuum region with a radius of 1.2m, where the outer 0.5m is a perfectly matched layer (PML), so there are no reflected light returning form the boundary. The background field excitations are used as incident wave. Besides, according to equation (3), there is a circle with radius $r_0 = 0.2m$ as the path of integration, the electric fields on the path are exported to calculate the scattering coefficients.

7 The physical informed neural network

Deep neural network (DNN) is a data-driven method to fit the high-dimensional non-linear functions. After training, the DNN has a strong ability to dig out the common properties among these data points. That's the reason why the output of DNN can satisfy these potential physical laws. Meanwhile, utilizing the known fundamental laws during training process can also improve the DNN accuracy.

The physical informed neural network (PINN) is a type of neural network architecture which incorporates physical laws and constraints into its training process. PINNs have been used in a variety of applications, which can also be beneficial to improve the DNN accuracy in our research. If we add the physical laws to the loss function:

$$L(\theta) = MSE + \alpha L_{laws}, \tag{38}$$

where L_{laws} is cost function that measures whether the output satisfies the physical laws, and α is a weight factor. In the training process, as the loss gradually decreases, the neural network will automatically satisfy these laws. This method will help the DNN improve the accuracy when the training data sets are small, e.g., we have tried training the origin DNN and the PINN with 1800 training data points and 200 validation data points, the MSE between the predicted results and the ground truth are plotted in Figure S2(a), the MSE loss of PINN is significantly lower than the origin NN. However, the results are both unsatisfactory (notice the order of magnitude of MSE) because of the small data set, we need to increase the amount of the data to get a more precise NN. When the data quantity reached 3600, the difference between the two approaches almost disappeared (Figure S2(b)). And finally, when we input 36000 data to get the final precise result, the MSE distributions are similar (Figure S2(c)). Therefore, our conclusion is that in our study, when the data size is small and the required accuracy is not high, PINN is a very suitable method; but when the data size is large or high accuracy is required, PINN does not have a significant advantage.

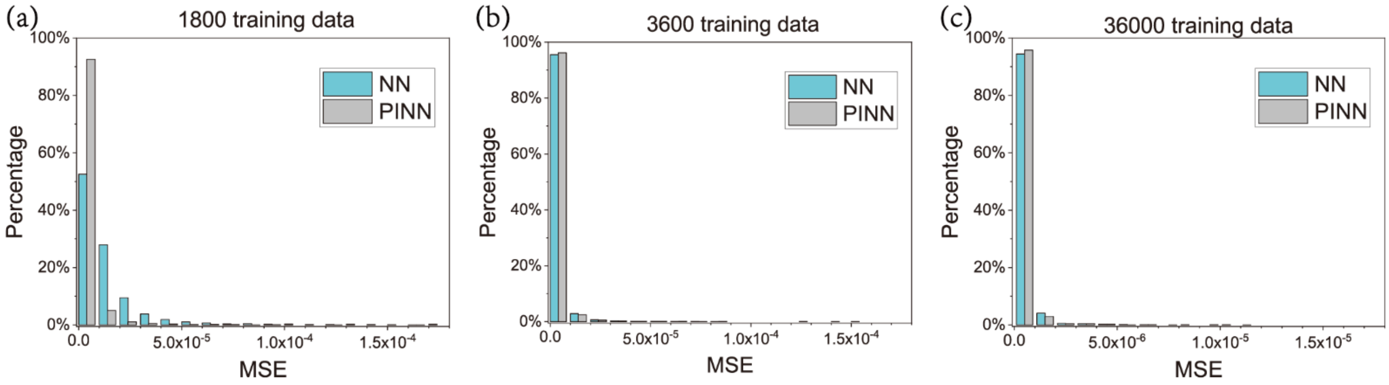

Figure S2. The testing data sets' MSE when the training data sets are equal to (a) 1800, (b) 3600 and (c) 36000.

8 The discussion about extending the DNN's range of application

Although the DNN promoted in the main text applies only to a hexagonal scatterer, it in principle could be extended to other frequency regimes and other shapes of scatterers or clusters, here we'd like to give some technical discussions.

Firstly, let's consider the polar coordinate system with the origin inside the scatterer. Any point on the boundary of the scatterer can be represented by (θ, ρ). We can approximate the boundary by quantizing the angel $\hat{\theta} = \lfloor \theta/\delta \rfloor \cdot \delta$, where δ is a small angle that control the degree of approximation. Then the boundary can be approximately represented as a $\lceil 2\pi/\delta \rceil$ dimensional vector of $[\rho_1, \rho_2, \ldots]$. Then the training and prediction routines detailed in our manuscript can be used with trivial adaption.

When it comes to a cluster contains k scatterers, the treatment is similar. The only technical issue is to have a parameterized representation of the boundaries of the k scatterers. A straight-forward method is

- We choose a fixed point as the global origin.
- We choose k 'origins', one for each scatterer. The local boundary representation for each scatterer is the same.
- The only addition is that we also need to encode the locations "local origins", thus adding 2k additional dimensions.
